# A positivity conjecture for a quotient of *q*-binomial coefficients

**DOI:** 10.1007/s11139-025-01285-2

**Published:** 2025-12-17

**Authors:** M. Gatzweiler, C. Krattenthaler

**Affiliations:** Fakultät für Mathematik, Universität Wien, Oskar-Morgenstern-Platz 1, 1090 Vienna, Austria

**Keywords:** *q*-binomial coefficients, Positivity, Rational *q*-Catalan polynomial, Primary 05A30, Secondary 33D05

## Abstract

We conjecture that, if the quotient of two *q*-binomial coefficients with the same top argument is a polynomial, then it has non-negative coefficients. We summarise what is known about the conjecture and prove it in two non-trivial cases. Moreover, we move ahead to extend our conjecture to D. Stanton’s fake Gaussian sequences. As a corollary of one of our results we obtain that a polynomial that is conjectured to be a cyclic sieving polynomial for Kreweras words [S. Hopkins and M. Rubey, *Selecta Math. (N.S.)* **28** (2022), Paper No. 10] is indeed a polynomial with non-negative integer coefficients.

We use the standard *q*-notations $[n]_q:=\frac{1-q^n}{1-q}$,

$$\begin{aligned} {[}n]_q!:=[n]_q\,[n-1]_q\cdots [1]_q, \quad \text {for }n\ge 1, \quad \text {and }[0]_q!:=1, \end{aligned}$$

and

$$\begin{aligned} \begin{bmatrix} n\\ k \end{bmatrix}_q:= {\left\{ \begin{array}{ll} \frac{[n]_q!}{[k]_q!\,[n-k]_q!},& \text {if }0\le k\le n,\\ 0,& \text {otherwise,} \end{array}\right. } \end{aligned}$$

to define *q*-*factorials* and *q*-*binomial coefficients*, respectively.

The purpose of this note is to put forward the following conjecture.

## Conjecture 1

Let *n*, *k*, *l* be non-negative integers. If

1 $$\begin{aligned} \frac{\begin{bmatrix} n\\ k \end{bmatrix}_q}{\begin{bmatrix} n\\ l \end{bmatrix}_q} =\frac{[l]_q!\,[n-l]_q!}{[k]_q!\,[n-k]_q!} \end{aligned}$$

is a polynomial (in *q*) then it has non-negative coefficients.

## Remark 2

(1) It is easy to see that it is enough to consider the range $1\le l<k\le \frac{n}{2}$.

(2) In evidence for this conjecture, we point out that it is trivially true for $l=k$ and $l=k-1$, well known for $l=0$ and $l=1$, implicitly known for $l=k-2$, and that we prove it for $l=k-3$ and $l=2$. These cases are treated in Cases 1–7 below and form the bulk of this paper. Moreover, we have verified Conjecture 1 for all $n\le 400$ with the help of *Mathematica*.

(3) As Sam Hopkins pointed out to us as a reaction on an earlier version of this paper, our Conjecture 1 has some overlap with an unpublished conjecture of Stanton on *fake Gaussian sequences* [1, Conj. 1]. It says:

*If*

2 $$\begin{aligned} \prod _{i=1} ^{n}\frac{(1-q^{m+i})^{a_i}}{(1-q^{i})^{a_i}} \end{aligned}$$

*is a polynomial for all positive integers* *m*, *where* *n* *is another positive integer, and* $(a_1,a_2,\dots ,a_n)$ *is a symmetric sequence of non-negative integers, then it has non-negative coefficients for all* *m*.

Assuming $k>l$, if in the expression in (2) we replace *n* by $n-k+l$, choose $m=k-l$ and $(a_1,a_2,\dots ,a_n) =(0^l,1^{n-k-l},0^l)$, where $\alpha ^s$ stands for $\alpha$ repeated *s* times, then we obtain (1).^1^ While it seems that our Conjecture 1 is a special case of the above conjecture, this is not so. Stanton’s conjecture requires polynomiality *for all* *m*, which is a big restriction. We do not do that. However, on the basis of numerous computations with “random” *m* and “random” sequences^2^$(a_1,a_2,\dots ,a_n)$ (see Remark 7.(2) after the proof of Lemma 6 for further evidence), we tend to believe that Stanton’s polynomiality restriction can be dropped and our weaker hypothesis is sufficient.

## Conjecture 3

Let *m* and *n* be given positive integers, and let $(a_1,a_2,\dots ,a_n)$ be a symmetric sequence of non-negative integers. If the expression (2) is a polynomial (in *q*) then it has non-negative coefficients.

We furthermore believe that even symmetry is not essential here.

## Conjecture 4

Let *m* and *n* be given positive integers with $m\ge n$, and let $(a_1,a_2, \dots ,a_n)$ be a sequence of non-negative integers. If the expression (2) is a polynomial (in *q*) then it has non-negative coefficients.

The restriction of $m\ge n$ should be noted. Stanton [1, bottom of p. 1] provides the example $m=1$ and $(a_1,\dots ,a_{17})=(1,3,1,1,1,1,1,1,2,1,1,1,1,1,1,1,1)$ where (2) is a polynomial that contains the term $-q^7$. However, computer calculations up to $m=200$ indicate that expression (2) with the same $a_i$’s is a polynomial (this was already shown by Stanton) with non-negative coefficients not only for all $m\ge 17$, but already for all $m\ge 2$.

(4) We use our result for $l=2$ to confirm a conjecture of Hopkins and Rubey [2] about a presumed cycling sieving polynomial; see Corollary 10 below.

(5) Conjecture 1 is reminiscent of Warnaar and Zudilin’s “*q*-rious positivity” [3]. However, it is not a special case since Landau’s criterion is never satisfied here (except in the trivial cases where $l=0$ or where $l=k$). On the other hand, it is also not true that Warnaar and Zudilin’s conjecture can be generalised to a result where one replaces Landau’s criterion by the assumption of polynomiality of the *q*-factorial expression. For example, we have

$$\begin{aligned} \frac{[12]_q!\,([2]_q!)^2}{[11]_q!\,[4]_q!\,[1]_q!}&=1 - q^2 + q^3 + q^4 - q^5 + q^7,\\ \frac{[10]_q!\,[4]_q!\,[3]_q!\,[1]_q!}{[9]_q!\,[5]_q!\,([2]_q!)^2}&=1 + q^2 - q^3 + q^4 + q^6,\\ \frac{[12]_q!\,([2]_q!)^3}{[11]_q!\,[4]_q!\,([1]_q!)^3}&=1 + q - q^2 + 2 q^4 - q^6 + q^7 + q^8,\\ \frac{[12]_q!\,([2]_q!)^5}{[11]_q!\,[4]_q!\,([1]_q!)^7}&=1 + 3 q + 2 q^2 - q^3 + q^4 + 4 q^5 + q^6 - q^7 \\&\quad + 2 q^8 + 3 q^9 + q^{10}. \end{aligned}$$

(6) The expressions in (1) and (2) are (if polynomial) clearly *cyclotomic generating functions* in the sense of [4].

As announced, we now discuss the special cases where $l=k,k-1,k-2,k-3$ and $l=0,1,2$.

Case 1: $l=k$. This is trivial since the quotient in (1) reduces to 1.

Case 2: $l=k-1$. In that case, the quotient in (1) reduces to

$$\begin{aligned} \frac{[n-k+1]_q}{[k]_q} =\frac{1-q^{n-k+1}}{1-q^k}. \end{aligned}$$

Clearly, this is a polynomial if and only if *k* divides $n-k+1$, and then it is a terminating geometric series, which has non-negative coefficients.

Case 3: $l=k-2$. Here, the quotient in (1) reduces to

3 $$\begin{aligned} \frac{[n-k+2]_q\,[n-k+1]_q}{[k]_q\,[k-1]_q} =\frac{(1-q^{n-k+2})(1-q^{n-k+1})}{(1-q^{k})(1-q^{k-1})}. \end{aligned}$$

At this point, we should recall that

4 $$\begin{aligned} q^n-1=\prod _{d\mid n} ^{}C_d(q), \end{aligned}$$

where $C_d(q)$ denotes the *d*-th cyclotomic polynomial in *q*, all of which are irreducible. Hence, the fraction (3) can only be a polynomial if both *k* and $k-1$ divide one of $n-k+1$ or $n-k+2$. If one of them divides $n-k+1$ and the other divides $n-k+2$, then we are back in the situation of Case 2, confirming the assertion of Conjecture 1.

It remains to discuss the case where both *k* and $k-1$ divide the same number, that is, either $n-k+1$ or $n-k+2$. In order to cope with this situation, we quote [5, Cor. 6]; see also [6, Ex. 1.8].

## Lemma 5

Let *a* and *b* be coprime positive integers, and let $\gamma$ be an integer with $\gamma \ge (a-1)(b-1)$. Then the expression

$$\begin{aligned} \frac{[ab]_q\,[\gamma ]_q}{[a]_q\,[b]_q} \end{aligned}$$

is a polynomial in *q* with non-negative integer coefficients.

Now, if we suppose that both *k* and $k-1$ divide $n-k+2$, say $n-k+2=mk(k-1)$, then the fraction (3) can be written in the form

$$\begin{aligned} \frac{[k(k-1)]_q\,[n-k+1]_q}{[k]_q\,[k-1]_q} \cdot \frac{[mk(k-1)]_q}{[k(k-1)]_q}. \end{aligned}$$

By Lemma 5, the first term in this product is a polynomial with non-negative coefficients, while the second term is again a terminating geometric series.

The case where both *k* and $k-1$ divide $n-k+1$ is treated in the same manner.

Altogether, this confirms Conjecture 1 in the current case.

Case 4: $l=k-3$. For this choice of *l*, the fraction (1) becomes

5 $$\begin{aligned} \frac{[n-k+3]_q\,[n-k+2]_q\,[n-k+1]_q}{[k]_q\,[k-1]_q\,[k-2]_q} =\frac{(1-q^{n-k+3})(1-q^{n-k+2})(1-q^{n-k+1})}{(1-q^{k})(1-q^{k-1})(1-q^{k-2})}. \end{aligned}$$

First of all, by the argument using cyclotomic polynomials that we already used in Case 3, for (5) to be a polynomial, *k*, $k-1$, and $k-2$ must each divide one of the arguments of the numerator factors, that is, one of $n-k+3$, $n-k+2$, or $n-k+1$. Following the arguments of Case 3, we must now distinguish between several cases.

It might be that *k*, $k-1$, and $k-2$ each divide a different factor^3^ from the numerator. Then we are again back in the situation of Case 2, confirming the assertion of Conjecture 1.

It might be that two of *k*, $k-1$, and $k-2$ divide the same factor in the numerator, while the remaining one divides a different factor. If, say, *k* and $k-1$ divide the same factor in the numerator, while $k-2$ divides a different one, then Lemma 5 applies again and shows that we obtain a polynomial with non-negative coefficients. The other case is when *k* and $k-2$ divide the same factor in the numerator, while $k-1$ divides a different one. This case has two subcases. If *k* and $k-2$ are relatively prime, then we argue as before and see, using again Lemma 5, that we obtain a polynomial with non-negative coefficients. However, it might be that 2 divides both *k* and $k-2$. Counting cyclotomic factors on top and in the bottom of (5), we infer that there must also be two terms out of $n-k+3$, $n-k+2$, and $n-k+1$ that are divisible by 2. Clearly, these must be $n-k+3$ and $n-k+1$. If $k-1$ divides $n-k+2$, then another application of Lemma 5 leads to the desired conclusion of non-negativity of coefficients of the polynomial (5). If not, then the situation is more complicated, see the next paragraph.

Continuing the previous case, we suppose that *k* and $k-2$ are even and divide $n-k+1$, and that $k-1$ divides $n-k+3$. Let $k=2K$ and $n-k+1=m\frac{k(k-2)}{2}=mK(2K-2)$ for appropriate positive integers *K* and *m*. Since $k-1$ divides $n-k+3$, we must have

$$\begin{aligned} m\frac{k(k-2)}{2}+2 =2K(K-1)m+2\equiv 0~\text {(mod }2K-1\text {)}. \end{aligned}$$

After simplification, we obtain the congruence

$$\begin{aligned} (K-1)m\equiv -2~\text {(mod }2K-1\text {)}. \end{aligned}$$

Since 2 and $2K-1$ are relatively prime, we may multiply both sides of the congruence by 2 and get

$$\begin{aligned} 2(K-1)m\equiv -4~\text {(mod }2K-1\text {)}, \end{aligned}$$

or, after simplification,

$$\begin{aligned} m\equiv 4~\text {(mod } 2K-1\text {)}. \end{aligned}$$

Consequently, we may write $m=4+M(2K-1)$, for some non-negative integer *M*. If we substitute these parametrisations in (5), then we obtain the fraction in (6) below. In Lemma 6 (also below) it is shown that this fraction is a polynomial with non-negative coefficients.

In the other case, if *k* and $k-2$ are even but divide $n-k+3$ while $k-1$ divides $n-k+1$, one can proceed similarly. One finally arrives at the fraction in (7), and in Lemma 6 it is shown that this fraction is also a polynomial with non-negative coefficients.

Finally, we turn to the case where all of *k*, $k-1$, and $k-2$ divide the same number out of $n-k+3$, $n-k+2$, and $n-k+1$. If *k* is odd, then *k*, $k-1$, and $k-2$ are pairwise coprime. Let us write $A_1,A_2,A_3$ for $n-k+3,n-k+2,n-k+1$, in some order, such that *k*, $k-1$, and $k-2$ divide $A_1$. Using this notation, we may rewrite the fraction in (5) in the form

$$\begin{aligned} \frac{[k(k-1)]_q\,[A_2]_q}{[k]_q\,[k-1]_q}\cdot \frac{[k(k-1)(k-1)]_q\,[A_3]_q}{[k(k-1)]_q\,[k-2]_q}\cdot \frac{[A_1]_q}{[k(k-1)(k-2)]_q}. \end{aligned}$$

Lemma 5 applies to the first two terms in this product, while the third term is a terminating geometric series. This shows that the above fraction is a polynomial with non-negative coefficients.

On the other hand, if *k* is even then, arguing as before, also $n-k+1$ and $n-k+3$ must be even. We use again the earlier notation, with *k*, $k-1$, $k-2$ all dividing $A_1$. In this case, we write the fraction in (5) in the form

$$\begin{aligned} \frac{[k(k-2)/2]_q\,[A_2]_q}{[k]_q\,[k-2]_q}\cdot \frac{[k(k-1)(k-2)/2]_q\,[A_3]_q}{[k(k-2)/2]_q\,[k-1]_q}\cdot \frac{[A_1]_q}{[k(k-1)(k-2)/2]_q}, \end{aligned}$$

where, here, $A_1$ and $A_2$ are chosen to be $n-k+1$ and $n-k+3$, in the appropriate order. Lemma 5 with *q* replaced by $q^2$ applies to the first term, while Lemma 5 applies directly to the second term. The third term is again a terminating geometric series.

Altogether, this confirms Conjecture 1 in our current case, Case 4, modulo the subsequent lemma.

## Lemma 6

Let *K* and *M* be non-negative integers. Then the fractions

6 $$\begin{aligned} \frac{ \prod _{i=0} ^{2}[4K(2K-2)+MK(2K-1)(2K-2)+i]_q}{[2K]_q\,[2K-1]_q\,[2K-2]_q},\quad \text {for }K\ge 2, \end{aligned}$$

and

7 $$\begin{aligned} \frac{ \prod _{i=0} ^{2}[K(2K-5)(2K-2)+MK(2K-1)(2K-2)-i]_q}{[2K]_q\,[2K-1]_q\,[2K-2]_q},\quad \text {for }K\ge 3, \end{aligned}$$

are polynomials with non-negative coefficients.

## Proof

We treat the first fraction. The second can be handled in a similar manner.

First it should be observed that the factors in the numerator of (6) are explicitly

$$\begin{aligned}  &   \left[ 2K(K-1)\big (4+M(2K-1)\big )\right] _q,\ \left[ 2K(K-1)\big (4+M(2K-1)\big )+1\right] _q,\\  &   \left[ 4K(2K-2)+MK(2K-1)(2K-2)+2\right] _q\\  &   \quad = \left[ 2(2K-1)\big ((2K-1)+MK(K-1)\big )\right] _q, \hspace{-1.0pt}\end{aligned}$$

so that, indeed, 2*K* and $2K-2$ divide the argument of the first factor, while $2K-1$ divides the argument of the third factor. Thus, all the cyclotomic polynomials $C_d(q)$ with $d\ge 2$ dividing $1-q^{2K}$ divide the first factor, as well as all the cyclotomic polynomials $C_d(q)$ with $d\ge 2$ dividing $1-q^{2K-2}$. There is one overlap though, namely $C_2(q)=1+q$ divides both $1-q^{2K}$ and $1-q^{2K-2}$, but only once the first factor. This is balanced by the third factor, which is also divisible by $1+q$. Since all the cyclotomic polynomials $C_d(q)$ with $d\ge 2$ dividing $1-q^{2K-1}$ divide the third factor — and $C_2(q)=1+q$ is not among them since $2K-1$ is odd — we have shown that the fraction (6) is a polynomial.

In order to make the following step more transparent, let us temporarily put

$$\begin{aligned} N_1&=(K-1)\big (4+M(2K-1)\big ),\\ N_2&=4K+MK(2K-1),\\ N_3&=2\big ((2K-1)+MK(K-1)). \end{aligned}$$

Using this notation, the fraction (6) can be written as

$$\begin{aligned}  &   \frac{ [2KN_1]_q\,[(2K-2)N_2+1]_q\,[(2K-1)N_3]_q}{[2K]_q\,[2K-1]_q\,[2K-2]_q}\\  &   =\left( 1-q^{(2K-2)N_2+1}\right) \cdot \frac{1-q^{2KN_1}}{1-q^{2K}}\cdot \frac{1}{1-q^{2K-2}}\cdot \frac{1-q^{(2K-1)N_3}}{1-q^{2K-1}}. \end{aligned}$$

Expansion of geometric series turns this into

8 $$\begin{aligned}  &   \sum _{j_1=0}^{N_1-1}\sum _{j_2\ge 0}\sum _{j_3=0}^{N_3-1} q^{2Kj_1+(2K-2)j_2+(2K-1)j_3}\nonumber \\  &   \quad -\sum _{j_1=0}^{N_1-1}\sum _{j_2\ge 0}\sum _{j_3=0}^{N_3-1} q^{2Kj_1+(2K-2)j_2+(2K-1)j_3+(2K-2)N_2+1}. \end{aligned}$$

A subsum of the first sum is

$$\begin{aligned}  &   \sum _{j_1=0}^{N_1-1}\sum _{j_4\ge 0}\sum _{j_5=0}^{N_3-2} q^{2Kj_1+(2K-2)(j_4+N_2-1)+(2K-1)(j_5+1)}\\  &   =\sum _{j_1=0}^{N_1-1}\sum _{j_4\ge 0}\sum _{j_5=0}^{N_3-2} q^{2Kj_1+(2K-2)j_4+(2K-1)j_5+(2K-2)N_2+1}. \end{aligned}$$

We see that this largely cancels with the second sum in (8), where only the terms with $j_3=N_3-1$ remain. These form a series of order^4^

9 $$\begin{aligned}  &   (2K-1)(N_3-1)+(2K-2)N_2+1 \nonumber \\  &   \quad = 16K^2-18K+4+4MK(K-1)(2K-1). \end{aligned}$$

The total degree of the polynomial (as we now know) in (6) is

$$\begin{aligned} 24K(K-1)+6MK(K-1)(2K-1)-6K+6. \end{aligned}$$

The order in (9) is larger than half of this degree. Hence, by (8) and the above considerations, we have shown that the polynomial in (6) has non-negative coefficients at least up to terms of half of the degree of the polynomial. Since the polynomial in (6) is a reciprocal polynomial^5^ it follows that also the other coefficients must be non-negative. $\square$

## Remark 7

(1) Clearly, we could continue in this manner and next consider the case where $l=k-4$, etc. However, it is equally clear that the corresponding arguments get more and more involved. Since we did not see how to build a general reasoning that would work for any *l* and *k*, we stop these considerations at this point and turn to cases where the value of *l* is small.

(2) Equipped with the previous arguments, we may also verify further special cases of Conjecture 3.

If the sequence $(a_1,a_2,\dots ,a_n)$ is of the form $(0^s,a,0^s)$ for some non-negative integers *a* and *s*, then, by taking the *a*-th root of expression (2), we see that we are back in Case 2. Similarly, if the sequence $(a_1,a_2,\dots ,a_n)$ is of the form $(0^s,a,a,0^s)$, then we are back in Case 3.

If the sequence $(a_1,a_2,\dots ,a_n)$ is of the form $(0^s,a,b,a,0^s)$, then we see that the arguments in Case 4 can be almost straightforwardly adapted to cover this slightly more general case as well.

Case 5: $l=0$. Then the quotient in (1) reduces to the *q*-binomial coefficient $$\left[ \begin{smallmatrix}n\\ k \end{smallmatrix}\right] _q$$. It is well-known that this has non-negative coefficients, see e.g. [7, Theorem 3.2].

Case 6: $l=1$. Then the quotient in (1) reduces to $$\frac{1}{[n]_q}\left[ \begin{smallmatrix}n\\ k \end{smallmatrix}\right] _q$$. If $\gcd (k,n-k)=1$, this is known as a *rational q-Catalan polynomial*, see e.g. [8, Sec. 5]. It is known that it has non-negative coefficients. However, since we were not able to find a proof of the *equivalence* that $$\frac{1}{[n]_q}\left[ \begin{smallmatrix}n\\ k \end{smallmatrix}\right] _q$$ is a polynomial in *q* *if and only if* $\gcd (k,n-k)=1$ in the literature, for completeness and for the convenience of the reader we provide a proof of all these facts.

## Theorem 8

The rational function

10 $$\begin{aligned} \frac{1}{[n]_q}\left[ \begin{matrix} n\\ k \end{matrix}\right] _q \end{aligned}$$

is a polynomial if and only if $\gcd (k,n-k)=1$. In the latter case, it is a polynomial with non-negative integer coefficients.

## Proof

By (4), the rational function in (10) can be decomposed into a product of cyclotomic polynomials (with positive and negative exponents). The cyclotomic polynomial $C_d(q)$ appears in (10) with exponent

11 $$\begin{aligned} \left\lfloor \tfrac{n-1}{d}\right\rfloor -\left\lfloor \tfrac{k}{d}\right\rfloor -\left\lfloor \tfrac{n-k}{d}\right\rfloor +\chi (d=1). \end{aligned}$$

Here, $\chi ({\mathcal {A}})=1$ if ${\mathcal {A}}$ is true and $\chi ({\mathcal {A}})=0$ otherwise. We must show that this is non-negative for all $d\ge 1$ if and only if $\gcd (k,n-k)=1$.

We first note that (11) vanishes for $d=1$. From now on, let $d\ge 2$. Writing $N=\left\{ \frac{n-1}{d}\right\}$ and $K=\left\{ \frac{k}{d}\right\}$, where $\{\alpha \}$ denotes the fractional part of a real number $\alpha$, we may convert (11) into

12 $$\begin{aligned} \left\lfloor N\right\rfloor -\left\lfloor K\right\rfloor -\left\lfloor N-K+\tfrac{1}{d}\right\rfloor =-\left\lfloor N-K+\tfrac{1}{d}\right\rfloor . \end{aligned}$$

This is non-negative except when $N-K+\tfrac{1}{d}=1$. That equation however is equivalent with $N=\frac{d-1}{d}$ and $K=0$. This means that $d\mid n$ and $d\mid k$, which implies $\gcd (k,n-k)>1$. This proves the first assertion in the statement of the theorem.

Let $\gcd (k,n-k)=1$. For the proof of the second assertion, we reproduce an argument that can be found in [9, Theorem 2] or [10, Prop. 10.1(iii)]. We recall that it is well-known that the *q*-binomial coefficient $$\left[ \begin{smallmatrix}n\\ k \end{smallmatrix}\right] _q$$ is a reciprocal and unimodal^6^ polynomial in *q* of degree $k(n-k)$. As a consequence, the expression

$$\begin{aligned} (1-q)\left[ \begin{matrix} n\\ k \end{matrix}\right] _q \end{aligned}$$

is a polynomial with a non-negative coefficient of $q^s$ for $0\le s\le \frac{1}{2}k(n-k)$. We multiply this expression by the geometric series $\frac{1}{1-q^n}=1+q^n+q^{2n}+\cdots$. It follows that

13 $$\begin{aligned} \frac{1-q}{1-q^n}\left[ \begin{matrix} n\\ k \end{matrix}\right] _q =\frac{1}{[n]_q}\left[ \begin{matrix} n\\ k \end{matrix}\right] _q \end{aligned}$$

is a polynomial (by the first part, since we assumed that $\gcd (k,n-k)$=1) with a non-negative coefficient of $q^s$ for $0\le s\le \frac{1}{2}k(n-k)$. Since also (13) is a reciprocal polynomial, all the other coefficients are non-negative as well. $\square$

Case 7: $l=2$. Then the quotient in (1) reduces to

14 $$\begin{aligned} \frac{[1]_q\,[2]_q}{[n]_q\,[n-1]_q}\begin{bmatrix} n\\ k \end{bmatrix}_q =\frac{(1-q)(1-q^2)}{(1-q^n)(1-q^{n-1})}\begin{bmatrix} n\\ k \end{bmatrix}_q. \end{aligned}$$

We show in the theorem below that, if this is a polynomial, it has non-negative coefficients.

## Theorem 9

The rational function (14) is a polynomial if and only if

15 $$\begin{aligned} \{\gcd (k,n-k),\gcd (k,n-k-1),\gcd (k-1,n-k)\}\subseteq \{1,2\}. \end{aligned}$$

In that case, it is a polynomial with non-negative integer coefficients.

## Proof

Let again $C_d(q)$ denote the *d*-th cyclotomic polynomial in *q*. By (4), the cyclotomic polynomial $C_d(q)$ appears in (14) with exponent

16 $$\begin{aligned} \left\lfloor \tfrac{n-2}{d}\right\rfloor -\left\lfloor \tfrac{k}{d}\right\rfloor -\left\lfloor \tfrac{n-k}{d}\right\rfloor +2\chi (d=1)+\chi (d=2). \end{aligned}$$

We must show that this is non-negative for all $d\ge 1$ if and only if the condition in (15) holds.

We begin with $d=1$: in that case (16) equals zero.

Next let $d=2$. In that case, the expression (16) reduces to

$$\begin{aligned} \left\lfloor \tfrac{n}{2}\right\rfloor -\left\lfloor \tfrac{k}{2}\right\rfloor -\left\lfloor \tfrac{n-k}{2}\right\rfloor . \end{aligned}$$

It is easy to see that this is always non-negative.

From now on, let $d\ge 3$. Writing $N=\left\{ \frac{n-2}{d}\right\}$ and $K=\left\{ \frac{k}{d}\right\}$, we may convert (16) into

17 $$\begin{aligned} \left\lfloor N\right\rfloor -\left\lfloor K\right\rfloor -\left\lfloor N-K+\tfrac{2}{d}\right\rfloor =-\left\lfloor N-K+\tfrac{2}{d}\right\rfloor . \end{aligned}$$

This is non-negative except if $N-K+\frac{2}{d}=1$ or $N-K+\frac{2}{d}=1+\frac{1}{d}$. These exceptions translate into the three cases

$$\begin{aligned} (N,K)\in \left\{ (\tfrac{d-2}{d},0),\, (\tfrac{d-1}{d},\tfrac{1}{d}),\, (\tfrac{d-1}{d},0)\right\} . \end{aligned}$$

If $(N,K)=(\tfrac{d-2}{d},0)$ then $d\mid n$ and $d\mid k$, implying $d\mid \gcd (k,n-k)$. This violates (15). If $(N,K)=(\tfrac{d-1}{d},\frac{1}{d})$ then $d\mid (n-1)$ and $d\mid (k-1)$, implying $d\mid \gcd (k-1,n-k)$. Again, this violates (15). Finally, if $(N,K)=(\tfrac{d-1}{d},0)$ then $d\mid (n-1)$ and $d\mid k$, implying $d\mid \gcd (k,n-k-1)$. This is also a violation of (15).

We have proved the first assertion in the statement of the theorem.

Now let the condition in (15) be satisfied. For the proof of the second assertion, we start with

18 $$\begin{aligned} \frac{1-q}{1-q^n}\begin{bmatrix} n\\ k \end{bmatrix}_q\quad \text {respectively} \quad \frac{1-q}{1-q^{n-1}}\begin{bmatrix} n-1\\ k-1 \end{bmatrix}_q, \end{aligned}$$

depending on whether $\gcd (k,n-k)=1$ or $\gcd (k,n-k)=2$. Given (15), both expressions in (18) are rational *q*-Catalan polynomials, which have even degree, as is easily seen. Furthermore, it follows from [12, Theorem 1.2 with $k=0$] that they are parity-unimodal^7^. Combined with the fact that they are also reciprocal polynomials, these observations imply that the products

$$\begin{aligned} (1-q^2)\times \frac{1-q}{1-q^n}\begin{bmatrix} n\\ k \end{bmatrix}_q\quad \text {respectively}\quad (1-q^2)\times \frac{1-q}{1-q^{n-1}}\begin{bmatrix} n-1\\ k-1 \end{bmatrix}_q \end{aligned}$$

have non-negative coefficients of $q^s$ for $s\le \frac{1}{2}\big (k(n-k)-(n-1)\big )$ respectively for $s\le \frac{1}{2}\big ((k-1)(n-k)-(n-2)\big )$. If we multiply the first expression by the geometric series $\frac{1}{1-q^{n-1}}$, respectively the second expression by $\frac{1}{1-q^k}$, we obtain the series

$$\begin{aligned} \frac{1-q^2}{1-q^{n-1}} \times \frac{1-q}{1-q^n}\begin{bmatrix} n\\ k \end{bmatrix}_q\quad \text {respectively}\quad \frac{1-q^2}{1-q^k} \times \frac{1-q}{1-q^{n-1}}\begin{bmatrix} n-1\\ k-1 \end{bmatrix}_q \end{aligned}$$

which have also non-negative coefficients of $q^s$ for $s\le \frac{1}{2}\big (k(n-k)-(n-1)\big )$ respectively for $s\le \frac{1}{2}\big ((k-1)(n-k)-(n-2)\big )$. However, both expressions equal the expression (14), of which we know that it is a polynomial. It is moreover a reciprocal polynomial, therefore also all other coefficients are non-negative. $\square$

As an application, we show that

19 $$\begin{aligned} \frac{ \prod _{j=1} ^{3n}(1-q^{2j})}{ \prod _{j=2} ^{2n+1}(1-q^j) \prod _{j=2} ^{n+1}(1-q^{2j})} \end{aligned}$$

is a polynomial with non-negative integer coefficients. This was conjectured by Hopkins and Rubey in [2, Conj. 6.2] as part of a presumed cyclic sieving phenomenon for Kreweras words.

## Corollary 10

The expression (19) is a polynomial with non-negative integer coefficients.

## Proof

For convenience, we introduce the usual notation for *q*-shifted factorials $(\alpha ;q)_m:=(1-\alpha )(1-\alpha q)\cdots (1-\alpha q^{m-1})$ for $m\ge 1$, and $(\alpha ;q)_0:=1$. Then (19) can be rewritten as

$$ \begin{aligned} \frac{(q^2;q^2)_{3n}}{(q^2;q)_{2n}\, (q^4;q^2)_n}&= \frac{(q^2;q^2)_{3n}\,(-q^2;q)_{2n}}{(q^4;q^2)_{2n}\,(q^4;q^2)_n} \& = \frac{(1-q^2)(1-q^4)\,(-q^3;q)_{2n-1}}{(1- q^{6n+4})(1-q^{6n+2})} \begin{bmatrix} 3n+2\\ n +1\end{bmatrix}_{q^2}. \end{aligned}$$

Using Theorem 9 with *q* replaced by $q^2$, *n* replaced by $3n+2$ and $k=n+1$, and the fact that $(-q^3;q)_{2n-1}$ is a polynomial in *q* with non-negative integer coefficients, we obtain the assertion of the corollary. $\square$

## Remark

The starting point for this work was indeed Conjecture 6.2 by Hopkins and Rubey [2]. Our investigations around this conjecture led us first to extract what has now become Theorem 9 as the essential germ. Later on we discovered the more general conjecture on quotients of *q*-binomial coefficients that is in the centre of this note.

## Data Availability

No datasets were generated or analysed during the current study.

## Footnotes

1 As we shall see in Case 5 below, for $l=0$ the expression in (1) reduces to a *q*-binomial coefficient, which is also known as *Gaußian polynomial* in the literature. Since, as we just argued, the expression (1) is a special case of the expression (2), the Gaußian polynomials are also subsumed by expression (2) as special cases. This explains Stanton’s notion of “fake Gaussian sequences.”

2 Aside from ad hoc “small number”-checks, we let several computers run for several days checking sequences $(0^s,a,b,b,a,0^s)$ and $(0^s,a,b,c,b,a,0^s)$, with *a*, *b*, *c*, *s* random integers between 0 and 10, and *m* between *s* and $1000+s$.

3 In abuse of language, we use abridged terminology here: “factor" should actually be “argument of a numerator factor".

4 The order of a formal power series in *q* is the smallest exponent *e* such that $q^e$ appears with non-zero coefficient in the series.

5 A polynomial *P*(*q*) in *q* of degree *m* is called *reciprocal* if $P(q)=q^mP(1/q)$.

6 A polynomial $P(q)= \sum _{i=0} ^{m}p_iq^i$ is called *unimodal* if there is an integer *r* with $0\le r\le m$ and $0\le p_0\le \dots \le p_r\ge \dots \ge p_m\ge 0$. It is well-known that *q*-binomial coefficients are unimodal; see [11, Ex. 7.75.d].

7 A polynomial $P(q)= \sum _{i=0} ^{m}p_iq^i$ is called *parity-unimodal* if the “even polynomial” $\sum _{i=0} ^{\left\lfloor m/2\right\rfloor }p_{2i}q^i$ and the “odd polynomial” $\sum _{i=0} ^{\left\lfloor (m-1)/2\right\rfloor }p_{2i+1}q^i$ are both unimodal.
